# Quantitative analysis of metal artifact reduction in total hip arthroplasty using virtual monochromatic imaging and orthopedic metal artifact reduction, a phantom study

**DOI:** 10.1186/s13244-021-01111-5

**Published:** 2021-11-24

**Authors:** Mark Selles, Vera H. Stuivenberg, Ruud H. H. Wellenberg, Loes van de Riet, Ingrid M. Nijholt, Jochen A. C. van Osch, Robbert W. van Hamersvelt, Tim Leiner, Martijn F. Boomsma

**Affiliations:** 1 Department of Radiology, Isala, 8025 AB Zwolle, The Netherlands; 2 Department of Radiology, Amsterdam University Medical Centre, 1105 AZ Amsterdam, The Netherlands; 3 Department of Medical Physics, Isala, 8025 AB Zwolle, The Netherlands; 4 Department of Radiology, University Medical Centre Utrecht, 3584 CX Utrecht, The Netherlands

**Keywords:** Arthroplasty, Replacement, Hip, CT, Dual energy

## Abstract

**Objective:**

To quantify metal artifact reduction using 130 keV virtual monochromatic imaging (VMI) with and without orthopedic metal artifact reduction (O-MAR) in total hip arthroplasty.

**Methods:**

Conventional polychromatic images and 130 keV VMI of a phantom with pellets representing bone with unilateral or bilateral prostheses were reconstructed with and without O-MAR on a dual-layer CT. Pellets were categorized as unaffected, mildly affected and severely affected.

**Results:**

When 130 keV VMI with O-MAR was compared to conventional imaging with O-MAR, a relative metal artifact reduction in CT values, contrast-to-noise (CNR), signal-to-noise (SNR) and noise in mildly affected pellets (67%, 74%, 48%, 68%, respectively; *p* < 0.05) was observed but no significant relative metal artifact reduction in severely affected pellets. Comparison between 130 keV VMI without O-MAR and conventional imaging with O-MAR showed relative metal artifact reduction in CT values, CNR, SNR and noise in mildly affected pellets (92%, 72%, 38%, 51%, respectively; *p* < 0.05) but negative relative metal artifact reduction in CT values and noise in severely affected pellets (− 331% and -223%, respectively; *p* < 0.05), indicating aggravation of metal artifacts.

**Conclusion:**

Overall, VMI of 130 keV with O-MAR provided the strongest metal artifact reduction.

**Supplementary Information:**

The online version contains supplementary material available at 10.1186/s13244-021-01111-5.

## Key points

- 130 keV VMI with O-MAR results in strongest metal artifact reduction.
- Only mild metal artifacts are reduced by 130 keV VMI without O-MAR.
- Severe metal artifacts are already reduced by conventional imaging with O-MAR.

## Background

Computed tomography (CT) is commonly used as imaging modality during postoperative follow-up after total hip arthroplasties (THA) [1]. CT is a relatively inexpensive, readily available modality and it is very effective in visualizing bone defects [2]. However, imaging of the pelvis in patients with large metal hip prostheses remains one of the biggest challenges in CT imaging since metallic prosthetic components lead to metal artifacts that make the assessment of surrounding bone and soft tissues difficult. Metal artifacts also hamper the detection of pathological capsular reactions of THA components [2]. Metal artifacts arise from beam hardening, scatter and photon starvation [2].

Several methods are available to reduce these metal artifacts. This includes basic steps such as increasing tube voltage and proper positioning of the patient. However, increasing tube voltage results in a higher required dose. More advanced methods include the use of model-based image reconstruction, iterative image reconstruction and metal artifact reduction (MAR) software [2, 3]. The artifact reduction ability of such software in conventional CT imaging of THA can be further increased by the addition of iterative model-based reconstruction (IMR) [1]. Another option to reduce artifacts is the use of dual-energy CT (DECT) with reconstruction of virtual monochromatic images at high energy levels. Prior work by Wellenberg and co-workers showed that the optimal monochromatic energy depends on the material of the prostheses. In general, virtual monochromatic imaging (VMI) at 130 keV was the optimal monochromatic energy level for metal artifact reduction in THA [4]. Several patient studies showed that the combination of MAR software and virtual monochromatic dual-energy CT imaging reduces metal artifacts even more effectively than one technique alone [5–7].

However, quantification of metal artifacts in patient studies is limited by anatomical differences between patients. By using a THA phantom, scans without hip prostheses can serve as a direct reference for scans with hip prostheses. This allows systematic assessment of metal artifacts and quantitative analysis which is not possible in patients with THA. Hitherto, a thorough, quantitative analysis of metal artifact reduction by combined use of DECT and orthopedic metal artifact reduction (O-MAR) in a THA phantom has not been performed. Hence, the aim of this study was to quantitatively compare the extent to which metal artifacts in a THA phantom can be reduced by 130 keV VMI with O-MAR, 130 keV VMI without O-MAR, conventional polychromatic imaging with O-MAR and conventional imaging without O-MAR.

## Methods

### Phantom

**Fig. 1:**
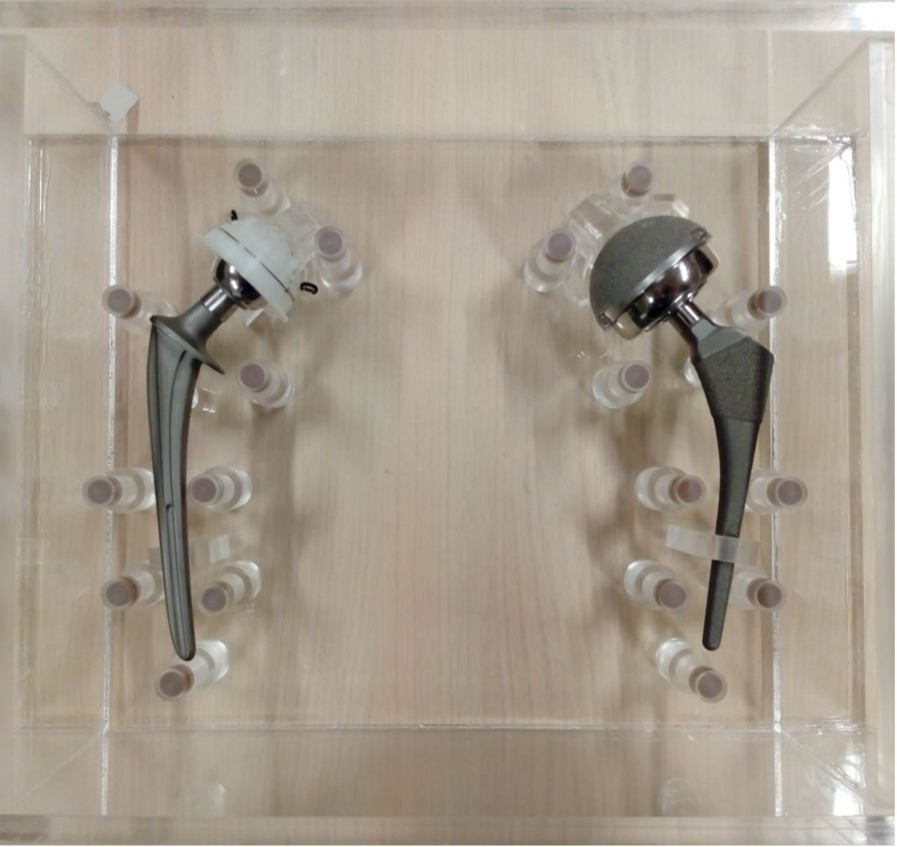
Image of the phantom containing bilateral prostheses at the right (cup: UHMWPE, head: ZTA, stem: CoCrMo) and left (cup: CoCrMo, head: CoCrMo, stem: TiAlV). *UHMWPE* ultrahigh molecular weight erthylene, *ZTA* Zirconia toughened aluminum, *TiAlV* titanium–aluminum–vanadium, *CoCrMo* cobalt–chrome–molybdenum

**Fig. 2:**
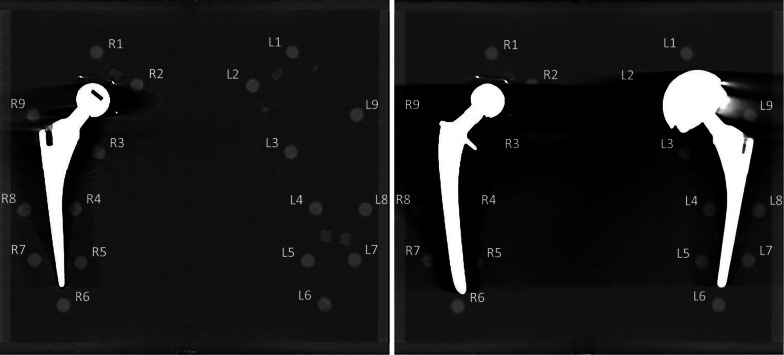
140 kVp polychromatic images of the phantom. Left image: phantom with unilateral prosthesis at the right (cup: UHMWPE, head: ZTA, stem: TiAlV). Right image: phantom with bilateral prostheses at the right (cup: UHMWPE, head: ZTA, stem: CoCrMo) and left (cup: CoCrMo, head: CoCrMo, stem: TiAlV). *UHMWPE* ultrahigh molecular weight erthylene, *ZTA* Zirconia toughene aluminum, *TiAlV* titanium–aluminum–vanadium, *CoCrMo* cobalt–chrome–molybdenum

The THA phantom was made of polymethyl methacrylate (PMMA) with the following dimensions: 320 mm wide, 130 mm high and 290 mm deep. The phantom contained 18 cylindrical pellets with a height and diameter of 10 mm (Fig. 1). These hydroxyapatite/calcium carbonate pellets all represented the same modular bone density and CT values of bone. The nine pellets on each side of the phantom were located at the acetabulum zone (DeLee and Charnley zones, pellets 1 and 2) and the most relevant radiological zones of the femur (the Gruen zones, pellets 3–9) (Fig. 2) [8, 9]. The phantom was filled with water, and air bubbles were removed.

### Image acquisition and reconstruction

Polychromatic and virtual monochromatic CT images were acquired on a Philips IQon 128 slice dual-layer detector CT scanner*.* The scan protocol was similar to clinical practice with regard to clinical evaluation of hip prostheses, using a standard CT dose index (CTDI) of 20 mGy at 140-kVp (Table 1). The phantom was imaged with either a unilateral or bilateral total hip prosthesis (Fig. 2). Acquisitions without prostheses served as a reference. The materials used in the phantom with unilateral prosthesis were ultrahigh molecular weight erthylene (UHMWPE) as prosthesis cup, Zirconia toughened aluminum (ZTA) as prosthesis head and titanium–aluminum–vanadium (TiAlV) as prosthesis stem. The materials used in the phantom with bilateral prostheses were UHMWPE (cup), ZTA (head) and CoCrMo (stem) for the right prosthesis and CoCrMo (cup), CoCrMo (head) and TiAlV (stem) for the left prosthesis. The materials of the head and stem and the UHMWPE cup are the commonly used for THA procedures in The Netherlands [10]. Although not commonly used, we chose to include a CoCrMo cup which aggravates metal artifacts as our aim was to explore to which extent metal artifacts can be reduced by different reconstructions.

**Table 1:** Scan protocol

	Philips iQon 128 slice dual-layer detector CT
Energy	140 kVp
Collimation	64 × 0.625 mm
FOV	330 mm
Pitch	0.392
Matrix	512 × 512
Rotation time	0.75 s
Dose (CTDI)	20 mGy
Filter	IMR: sharp plus
	Spectral level: sharp (C)
Increment	0.46
Slice thickness	0.9 mm

**Fig. 3:**
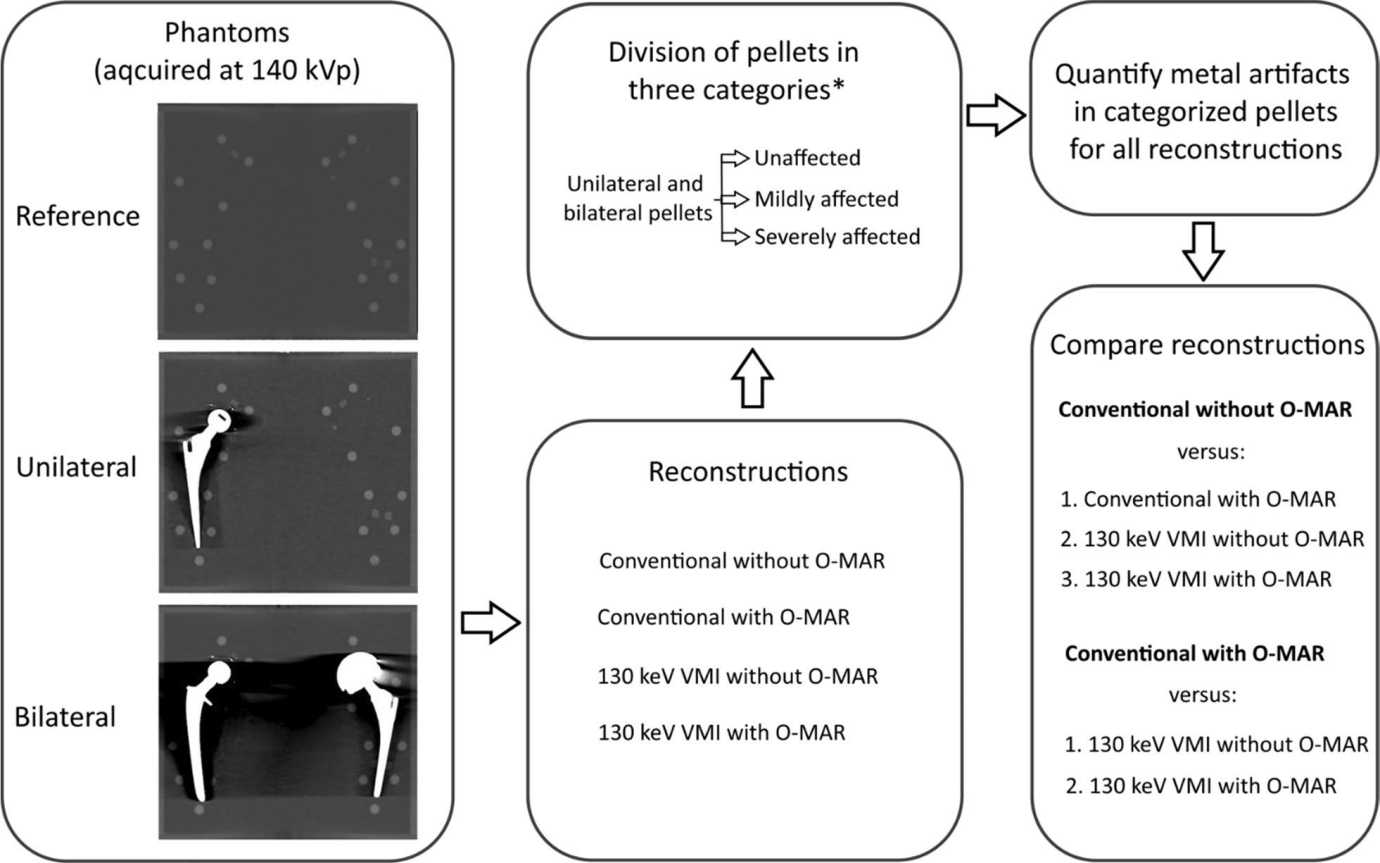
Study design. *Categorization of pellets was based on the conventional reference image without O-MAR

Figure 3 shows the design of this study. Polychromatic CT images acquired at 140 kVp were reconstructed using IMR level 1 and further referred to as ‘conventional’. Virtual monochromatic images (VMI) at 130 keV were extracted, since this keV resulted in optimal CNR in a similar previous study [4]. Both conventional images and 130 keV virtual monochromatic images were reconstructed with and without O-MAR (Philips Healthcare, Best, The Netherlands).

### Analysis of image quality

**Fig. 4:**
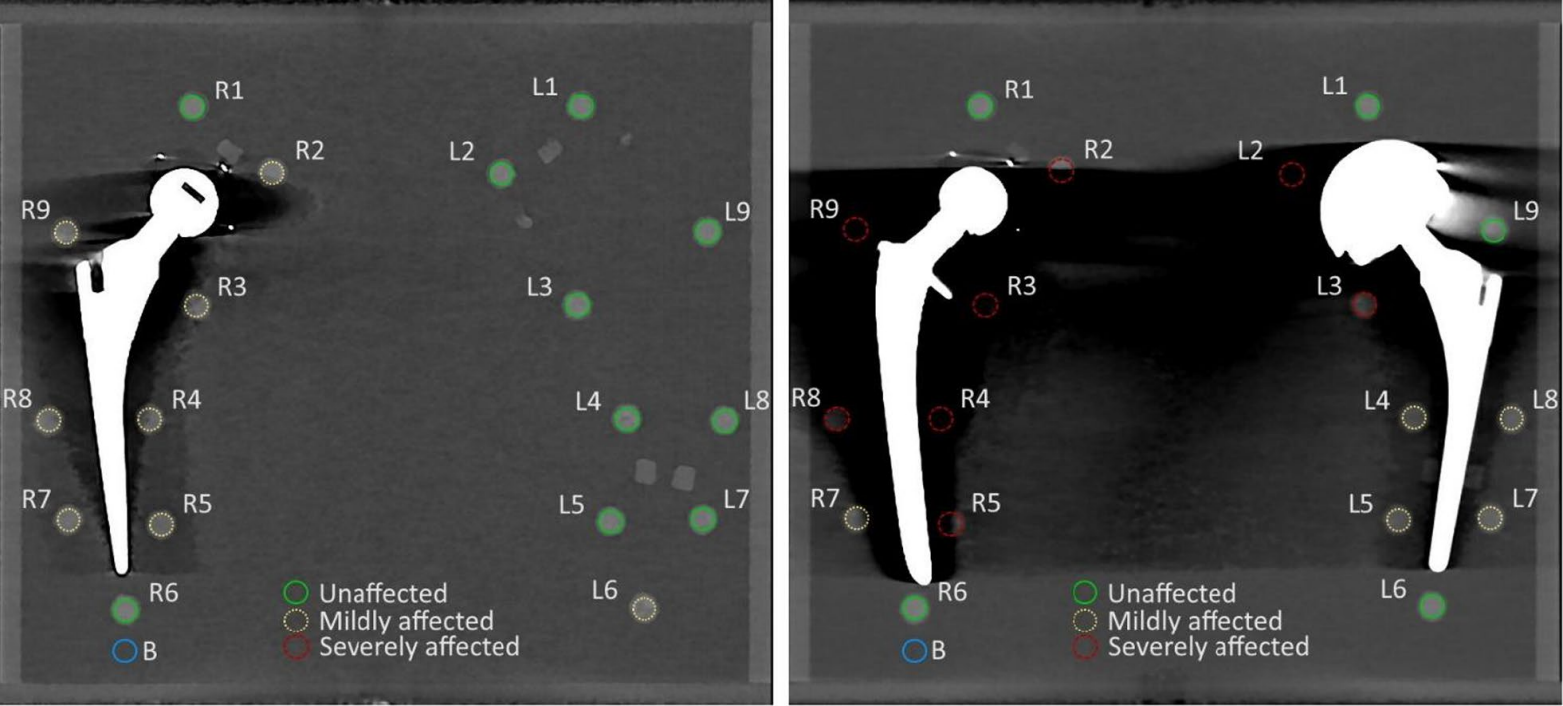
140 kVp conventional images without O-MAR with a unilateral prosthesis (left) and bilateral prostheses (right). Pellets were categorized as unaffected (green), mildly affected (yellow) and severely affected (red) pellets. The background region of interest (blue) was used to calculate contrast-to-noise ratio

For all obtained images, the coronal slice located in the middle of the prosthesis was extracted and used for quantitative analysis in ImageJ (1.48v). The images were analyzed by manually drawing nine regions of interest (ROIs) placed in the pellets at the left (L1-L9), nine ROIs placed in the pellets at the right (R1–R9) and one ROI in a homogeneous section of water which was unexposed to metal artifacts (Fig. 4). The diameter of the ROIs was 6.75 mm or 15 pixels to limit partial volume effects. To allow systematic analysis of the images, a standardized template of ROIs was used in ImageJ. The standardized template was used to measure CT numbers in Hounsfield units (HU) and the standard deviation in HU as a measure of noise. Signal-to-noise ratio (SNR) was calculated by dividing the HU by the noise of the pellets. Contrast-to-noise ratio (CNR) was calculated by dividing the difference in CT values between the pellet and background by the mean of the noise of the pellet and background noise.

**Fig. 5:**
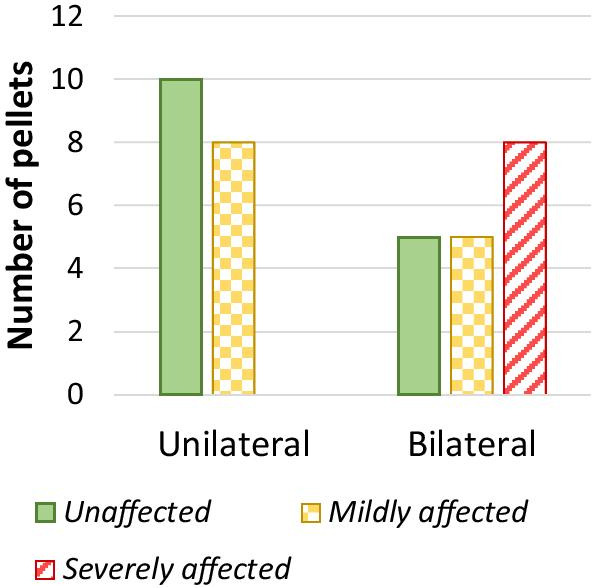
Number of pellets classified as unaffected, mildly affected or severely affected for unilateral and bilateral prostheses

Pellets were categorized as unaffected, mildly affected or severely affected based on the mean HU of the pellet in the unilateral and bilateral conventional images without O-MAR (Fig. 4). We chose to categorize the pellets to explore metal artifact reduction of mild and severe artifacts. Pellets with mean CT value < 0 HU were categorized as severely affected, pellets with mean CT value > 0 HU and CT value < 235 HU were categorized as mildly affected and pellets with CT value ≥ 235 HU were categorized as unaffected (Fig. 5). The cutoff value of 235 HU was determined using the conventional reference image without O-MAR. CT values < 235 HU were found to deviate more than the standard deviation from reference values and were therefore considered affected.

### Quantification of metal artifact reduction

The absolute difference in mean CT values, SNR, CNR or noise between the images with prostheses and reference images (without prostheses) was calculated for mildly and severely affected artifacts as a measure of metal artifact reduction. The difference was normalized as calculated by

1 $$\Delta V_{{{\text{reconstruction}}}} = \left| {1 - \frac{{V_{{{\text{with}}\;{\text{prostheses}}}} }}{{V_{{{\text{no}}\;{\text{prostheses}}}} }}} \right|_{{{\text{reconstruction}}}}$$

where *V *is either CT values, SNR, CNR or noise. Normalization was applied to allow comparison between the VMI and conventional images as the CT values on the VMI are notably lower due to the high virtual monochromatic energy.

The calculated differences for CT values, SNR, CNR and noise were compared for the reconstructions for mildly and severely affected artifacts to define the relative metal artifact reduction as follows:

2 $${\text{Relative metal artifact reduction}} \left( \% \right) = \left( {1 - \left( {\frac{{\Delta V_{{{\text{reconstruction}}}} }}{{\Delta V_{{{\text{standard reconstruction}}}} }}} \right)} \right)*100\%$$

where Δ*V* is calculated as shown in Eq. 1. Conventional imaging without O-MAR and conventional imaging with O-MAR were used as standard reconstructions in Eq. 2 because these images are commonly used in clinical practice. Relative metal artifact reduction was calculated to compare reconstructions. VMI of 130 keV with O-MAR, VMI of 130 keV without O-MAR and conventional imaging with O-MAR were compared to conventional imaging without O-MAR. VMI of 130 keV with O-MAR and VMI of 130 keV without O-MAR were compared to conventional imaging with O-MAR (Fig. 3).

### Statistical analysis

Wilcoxon signed-ranks tests were used to test for differences in metal artifact reduction between the reconstructions. A two-sided alpha of 5% was used as significance level. IBM SPSS software (version 23) was used for analysis.

## Results

Image quality parameters of conventional and VMI without O-MAR were different without insertion of prostheses (Table 2). Reconstructions with O-MAR resulted in the exact same values for these parameters as O-MAR does not change CT values when no metal is present.

**Table 2:** Mean image quality parameters for conventional 140 kVp and 130 keV virtual monochromatic imaging with and without O-MAR, without insertion of prostheses. Values are the mean of all 18 pellets ± standard deviation

	CT values (HU)	CNR	SNR	Noise (HU)
Conventional	248.4 ± 4.1	37.3 ± 2.8	36.5 ± 5.2	6.9 ± 1.0
Conventional with O-MAR	248.4 ± 4.1	37.3 ± 2.8	36.5 ± 5.2	6.9 ± 1.0
130 keV VMI	150.4 ± 4.5	18.0 ± 1.5	19.3 ± 3.2	8.0 ± 1.2
130 keV VMI with O-MAR	150.4 ± 4.5	18.0 ± 1.5	19.3 ± 3.2	8.0 ± 1.2

**Fig. 6:**
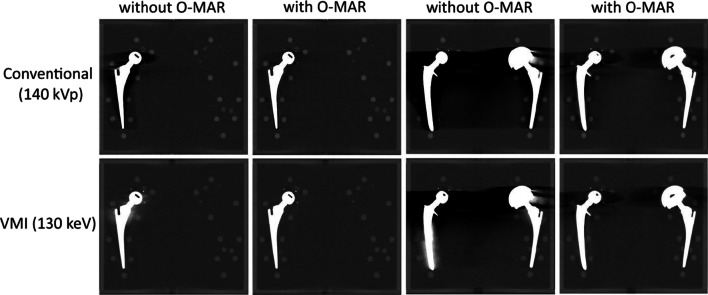
Coronal images of the phantom with unilateral or bilateral prostheses. Conventional images (upper row) and virtual monochromatic images (bottom row) were reconstructed with and without O-MAR. Unilateral images: phantom with prosthesis at the right (cup: UHMWPE, head: ZTA, stem: TiAlV). Bilateral images: phantom with prostheses at the right (cup: UHMWPE, head: ZTA, stem: CoCrMo) and left (cup: CoCrMo, head: CoCrMo, stem: TiAlV). *UHMWPE* ultrahigh molecular weight erthylene, *ZTA* Zirconia toughened aluminum, *TiAlV* titanium–aluminum–vanadium, *CoCrMo* cobalt–chrome–molybdenum

Conventional images of the phantom with bilateral protheses showed more streak artifacts compared to conventional images of the phantom with unilateral prosthesis. Conventional imaging with O-MAR resulted in a decrease of streak artifacts for both unilateral and bilateral images. VMI of 130 keV without O-MAR resulted in a decrease of streak artifacts, in particular for the unilateral prosthesis. However, bright streak artifacts were introduced on the 130 keV VMI. VMI of 130 keV with O-MAR reduced dark streak artifacts without introducing bright artifacts (Fig. 6).

**Fig. 7:**
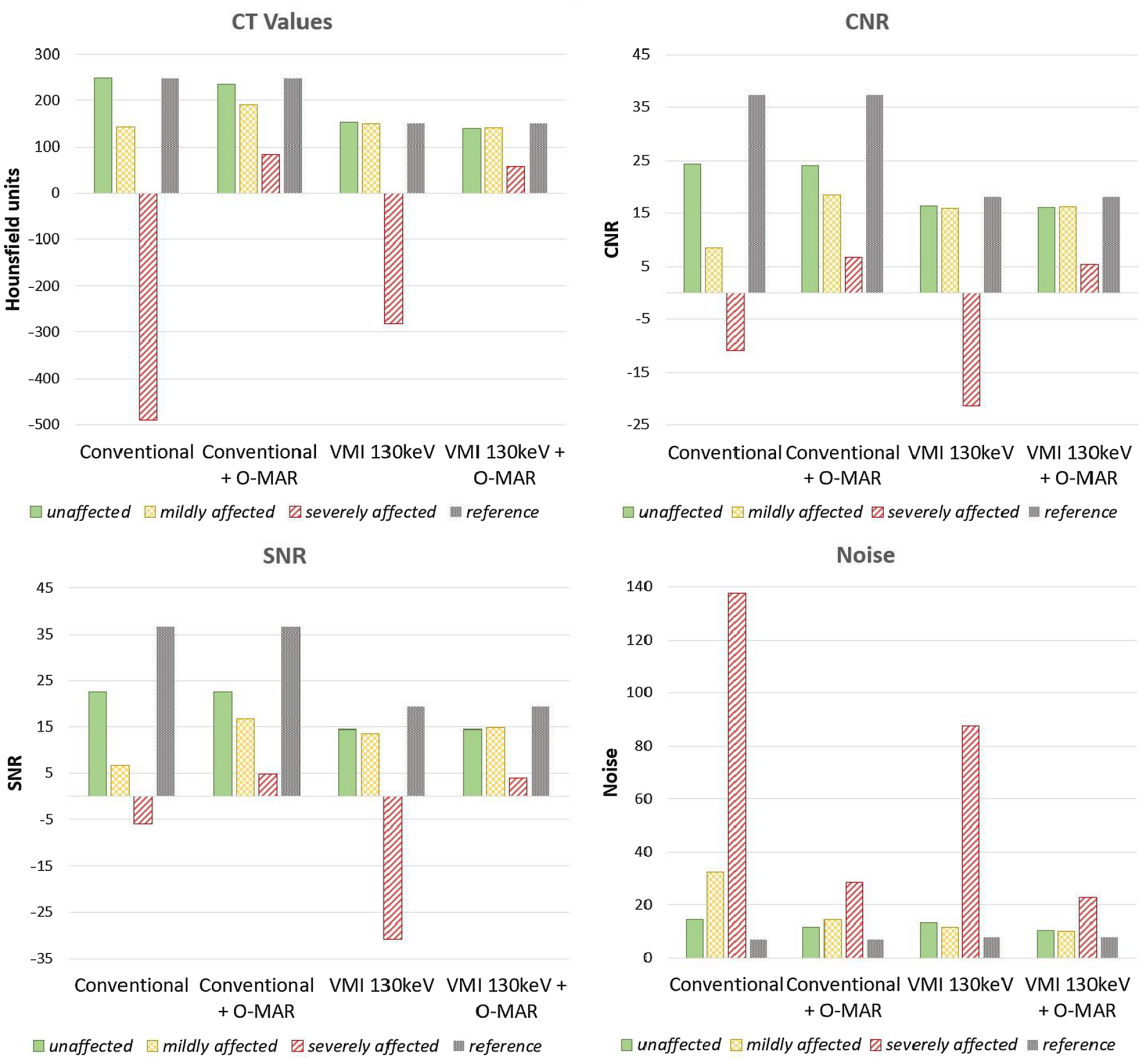
CT values in Hounsfield Units, contrast-to-noise ratio (CNR), signal-to-noise ratio (SNR) and noise for the categorized pellets for conventional (140 kVp) imaging with and without O-MAR and 130 keV virtual monochromatic imaging with and without O-MAR. Pellets were categorized in unaffected, mildly affected and severely affected pellets. Reference values are the average values of all the pellets in the phantom without insertion of prostheses

The highest CT values, CNR and SNR were found in conventional images with O-MAR for both mildly as severely affected pellets. The difference between both mildly and severely affected pellets and reference in CT values, CNR and SNR were lowest for 130 keV images with O-MAR. Also, the lowest noise was measured on the 130 keV images with O-MAR. VMI of 130 keV without O-MAR showed similar results to 130 keV images with O-MAR in CT values, CNR, SNR and noise for mildly affected pellets. However, CT values, CNR, SNR and noise were much lower and noise was much higher on the 130 keV images without O-MAR in comparison to 130 keV images with O-MAR for severely affected pellets (Fig. 7, see Additional file 1 for data of all individual pellets).

Conventional imaging with O-MAR resulted in significant metal artifact reduction in CT values, CNR, SNR and noise for mildly affected pellets (45%, 34%, 33% and 71%, respectively) and severely affected pellets (77%, 36%, 26% and 84%, respectively) in comparison to conventional imaging without O-MAR (Table 3). VMI of 130 keV without O-MAR resulted in significant metal artifact reduction for mildly affected pellets in CT values, CNR, SNR and noise (95%, 81%, 58% and 86%, respectively) and a significant metal artifact reduction of 47% in noise in severely affected artifacts in comparison to conventional imaging without O-MAR (Table 3). VMI of 130 keV with O-MAR also resulted in significant metal artifact reduction in CT values, CNR, SNR and noise for mildly affected pellets (81%, 83%, 66% and 91%, respectively) and severely affected pellets (79%, 46%, 32% and 90%, respectively) in comparison to conventional imaging without O-MAR (Table 3).

**Table 3:** Relative metal artifact reduction in CT values (HU), contrast-to-noise ratio, signal-to-noise ratio and noise in comparison to conventional (140kVp) images without O-MAR

	Metal artifact reduction (%) mildly affected pellets				Metal artifact reduction (%) severely affected pellets			
	HU	CNR	SNR	Noise	HU	CNR	SNR	Noise
Conventional + O-MAR	45% (*p* = 0.006)	34% (*p* = 0.002)	33% (*p* = 0.002)	71% (*p* = 0.002)	77% (*p* = 0.012)	36% (*p* = 0.012)	26% (*p* = 0.012)	84% (*p* = 0.012)
130 keV VMI	95% (*p* = 0.003)	81% (*p* = 0.001)	58% (*p* = 0.001)	86% (*p* = 0.001)	3% (*p* = 0.779)	–69% (*p* = 0.889)	–124% (*p* = 0.889)	47% (*p* = 0.012)
130 keV VMI + O-MAR	81% (*p* = 0.001)	83% (*p* = 0.001)	66% (*p* = 0.001)	91% (*p* = 0.001)	79% (*p* = 0.012)	46% (*p* = 0.012)	32% (*p* = 0.012)	90% (*p* = 0.012)

VMI of 130 keV without O-MAR resulted in significant metal artifact reduction in CT values, CNR and SNR in mildly affected pellets (92%, 72% and 38%, respectively) in comparison to conventional imaging with O-MAR but resulted in a significant decrease in metal artifact reduction in HU, SNR and noise in severely affected pellets (− 331%, − 202% and − 223%, respectively; Table 4). It should be noted that a negative metal artifact reduction indicates aggravation of the artifacts. VMI of 130 keV with O-MAR resulted in significant metal artifact reduction in CT values, CNR, SNR and noise for mildly (67%, 74%, 48% and 68%, respectively) in comparison to conventional imaging with O-MAR (Table 4).

**Table 4:** Relative metal artifact reduction in CT values (HU), contrast-to-noise ratio, signal-to-noise ratio and noise in comparison to conventional (140kVp) with O-MAR images

	Metal artifact reduction (%) mildly affected pellets				Metal artifact reduction (%) severely affected pellets			
	HU	CNR	SNR	Noise	HU	CNR	SNR	Noise
130 keV VMI	92% (*p* = 0.016)	72% (*p* = 0.005)	38% (*p* = 0.033)	51% (*p* = 0.249)	− 331% (*p* = 0.025)	− 166% (*p* = 0.093)	− 202% (*p* = 0.050)	− 223% (*p* = 0.036)
130 keV VMI + O-MAR	67% (*p* = 0.001)	74% (*p* = 0.002)	48% (*p* = 0.011)	68% (*p* = 0.033)	6% (*p* = 0.779)	15% (*p* = 0.208)	8% (*p* = 0.208)	39% (*p* = 0.093)

## Discussion

In this study, we quantified metal artifact reduction in a THA phantom using high energy VMI with and without O-MAR in comparison to conventional imaging with and without O-MAR. We found that 130 keV VMI with O-MAR resulted in the strongest metal artifact reduction of mild and severe metal artifacts.

A tube voltage of 140 kVp is often used to reduce metal artifacts and results in lower CT number inaccuracies, lower noise and higher SNR and CNR [11] in comparison to 120 kVp. However, increasing tube voltage may result in only a slight reduction of metal artifacts [12–17]. Moreover, a higher tube voltage leads to a higher required dose whereas extraction of VMI and application of O-MAR are not leading to a higher required dose.

In previous THA phantom studies, it was already reported that the use of O-MAR in conventional imaging reduces metal artifacts [1, 2, 18–21]. Our results confirmed the value of O-MAR in conventional imaging for metal artifact reduction. Although 130 keV VMI without O-MAR showed stronger metal artifact reduction for mildly affected pellets than conventional imaging with O-MAR, metal artifact reduction for severely affected pellets worsened in comparison to conventional imaging with O-MAR in our study. Besides, the metal artifact reduction in CT values of 92% when comparing 130 keV VMI without O-MAR and conventional imaging with O-MAR is probably heightened by the bright streak artifacts visible on the 130 keV VMI without O-MAR. Our finding that 130 keV VMI in only mildly affected pellets showed metal artifact reduction in comparison to conventional imaging may be explained by reduction of metal artifacts caused by beam hardening. VMI of 130 keV is less prone to beam hardening artifacts, which are dominant in mildly affected pellets, decreasing metal artifacts.

Severely affected pellets were only present in the images of the phantom with bilateral prostheses where photon starvation is dominant over beam hardening. These severe metal artifacts caused by bilateral implants were not reduced on 130 keV VMI without O-MAR, which is supported by previous studies [6, 11, 22]. On the other hand, conventional imaging with O-MAR did result in a reduction of metal artifacts in severely affected pellets. Similarly, 130 keV VMI with O-MAR resulted in a decrease of metal artifacts in severely affected pellets. In addition, 130 keV VMI with O-MAR resulted in a decrease of metal artifacts in mildly affected pellets compared to conventional imaging with O-MAR and therefore resulted in the strongest metal artifact reduction for both mildly as severely affected pellets.

Our quantitative analysis using a THA phantom shows that high energy VMI with O-MAR results in the strongest metal artifact reduction, and this is supported by studies incorporating subjective assessment by radiologists [5–7] and by studies incorporating quantitative assessment of metal artifact reduction [5, 7, 23]. However, these studies used General Electric (GE) or Siemens systems to extract VMI with metal artifact reduction software, while we used a Philips system to extract VMI with O-MAR. Although metal artifact reduction techniques of the different vendors show comparable results regarding metal artifact reduction using VMI and VMI with metal artifact reduction software, it remains difficult to compare the results. A phantom study comparing metal artifact reduction of the different vendors would provide more insight in the performance of the different DECT techniques of the vendors and their metal artifact reduction software.

Although 130 keV VMI with O-MAR resulted in the strongest relative metal artifact reduction, it should be noted that the CT values, CNR and SNR of the unaffected, mildly affected and severely affected pellets were lower in the 130 keV VMI with O-MAR in comparison to conventional images with O-MAR. This can be explained by the high virtual monochromatic energy of the 130 keV images, causing a reduction of the CT values in the images in comparison to conventional images. Although low SNR and CNR can affect the detectability of the pellets and therefore diligence is important, subjective analysis of patients shows that high energy VMI with O-MAR still can be assessed reliable [22]. Furthermore, the Rose criterion states that an object’s CNR must exceed 3–5 to be detectable [24, 25]. Both conventional imaging with O-MAR as well as 130 keV imaging with O-MAR exceeded this criterion for both mildly affected and severely affected pellets.

On our 130 keV images, bright artifacts were induced. Likewise, several studies described that VMI and metal artifact reduction software can induce secondary artifacts [7, 26, 27]. Hence, the radiologist should also review conventional images in addition to the images with metal artifact reduction. Furthermore, relatively high negative metal artifact reduction in CNR (− 69%) and SNR (− 124%) was observed in severely affected pellets while high *p* values (0.889 and 0.889, respectively) were found when comparing 130 keV VMI without O-MAR and conventional imaging without O-MAR, which seems contradictory. This may be explained by the large dispersion of CNR values and SNR values of the individual severely affected pellets in the 130 keV VMI without O-MAR, caused by the introduction of bright artifacts.

In this study, we focused on image quality in orthopedic prosthesis imaging. Hence, we used a phantom with pellets mimicking bone tissue and we did not assess soft tissue. However, assessment of structures such as the bladder, uterus and prostate is also hampered by metal artifacts, which is particularly important in oncology patients. A study to quantify metal artifact reduction using structures mimicking soft tissue will certainly be of interest*.*

Our study has limitations. First, we categorized the pellets in the images with the unilateral and bilateral prostheses in unaffected, mildly affected and severely affected which allowed comprehensible analysis of all pellets. However, mildly affected pellets were caused by unilateral and bilateral prostheses. Assignment of these pellets to one category may not be in accordance with clinical practice because a unilateral prosthesis causes different artifacts than bilateral prostheses Furthermore, pellet L9 in the bilateral image was categorized as unaffected despite bright streaking artifacts, and a more appropriate category may be mildly affected instead. Third, our results did not quantify metal artifact reduction in the close vicinity of the implant. Fourth, the pellets only represent one bone density, comparable to trabecular bone. Bone structures with higher densities such as cortical bone may alter the results. Finally, we chose to place the background ROI in a part of the image where no metal artifacts were present. Although this avoids metal artifacts in the background ROI, the calculated CNR does not quantify contrast between pellets and the background in the vicinity of the pellets.

## Conclusions

We conclude that 130 keV VMI with O-MAR shows stronger metal artifact reduction over 130 keV without O-MAR and conventional imaging with O-MAR. It should be encouraged to reconstruct high energy VMI with O-MAR besides conventional polychromatic images after THA.

## Supplementary Information

**Additional file 1.** CT values, contrast-to-noise ratios, signal-to-noise ratios and noise values of all individual pellets for unilateral and bilateral protheses.

## Data Availability

The datasets used and/or analyzed during the current study are available from the corresponding author on reasonable request.

## Abbreviations

CNR: Contrast-to-noise ratio
CT: Computed tomography
DECT: Dual-energy computed tomography
HU: Hounsfield unit
IMR: Iterative model-based reconstruction
keV: Kiloelectronvolt
kVp: Kilovoltage peak
MAR: Metal artifact reduction
mGy: Milli-gray
O-MAR: Orthopedic metal artifact reduction
PMMA: Polymethyl methacrylate
ROI: Region of interest
SNR: Signal-to-noise ratio
THA: Total hip arthroplasty
TiAlV: Titanium–aluminum–vanadium
UHMWPE: Ultrahigh molecular weight erthylene
VMI: Virtual monochromatic imaging
ZTA: Zirconia toughened aluminum
