# Supplementary material for: Quantitative analysis of metal artifact reduction in total hip arthroplasty using virtual monochromatic imaging and orthopedic metal artifact reduction, a phantom study
Source: Insights Imaging. 2021 Nov 24;12:171. doi: 10.1186/s13244-021-01111-5 (PMC8613319; doi:10.1186/s13244-021-01111-5)
Supplement: Supplementary file 1 — Additional file 1. CT values, contrast-to-noise ratios, signal-to-noise ratios and noise values of all individual pellets for unilateral and bilateral protheses. [file 13244_2021_1111_MOESM1_ESM.docx]

**ELECTRONIC SUPPLEMENTARY MATERIAL**


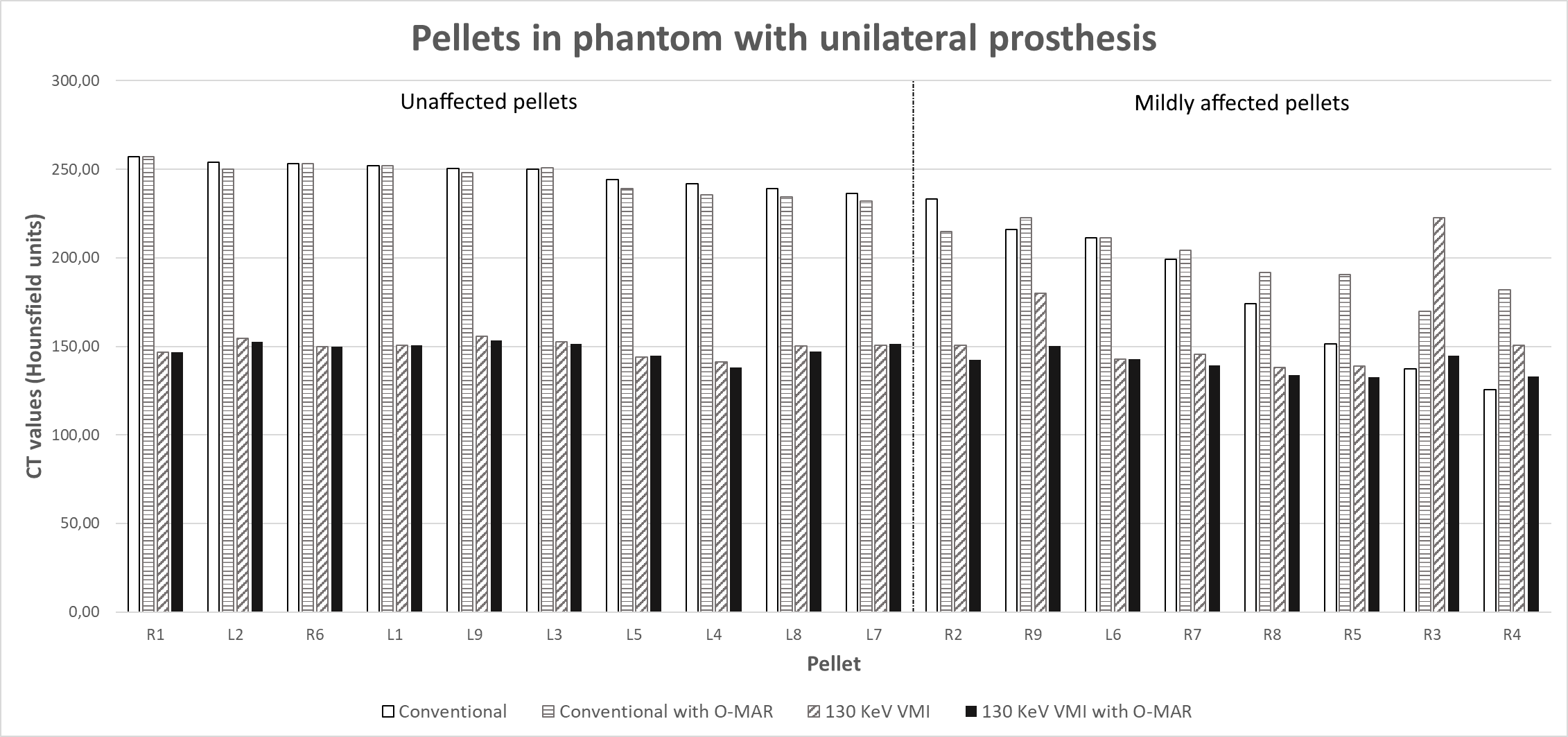


**Supplementary materials 1:** CT values in Hounsfield units of all pellets in the phantom with unilateral prosthesis. Unaffected pellets at the left, mildly affected pellets at the right.


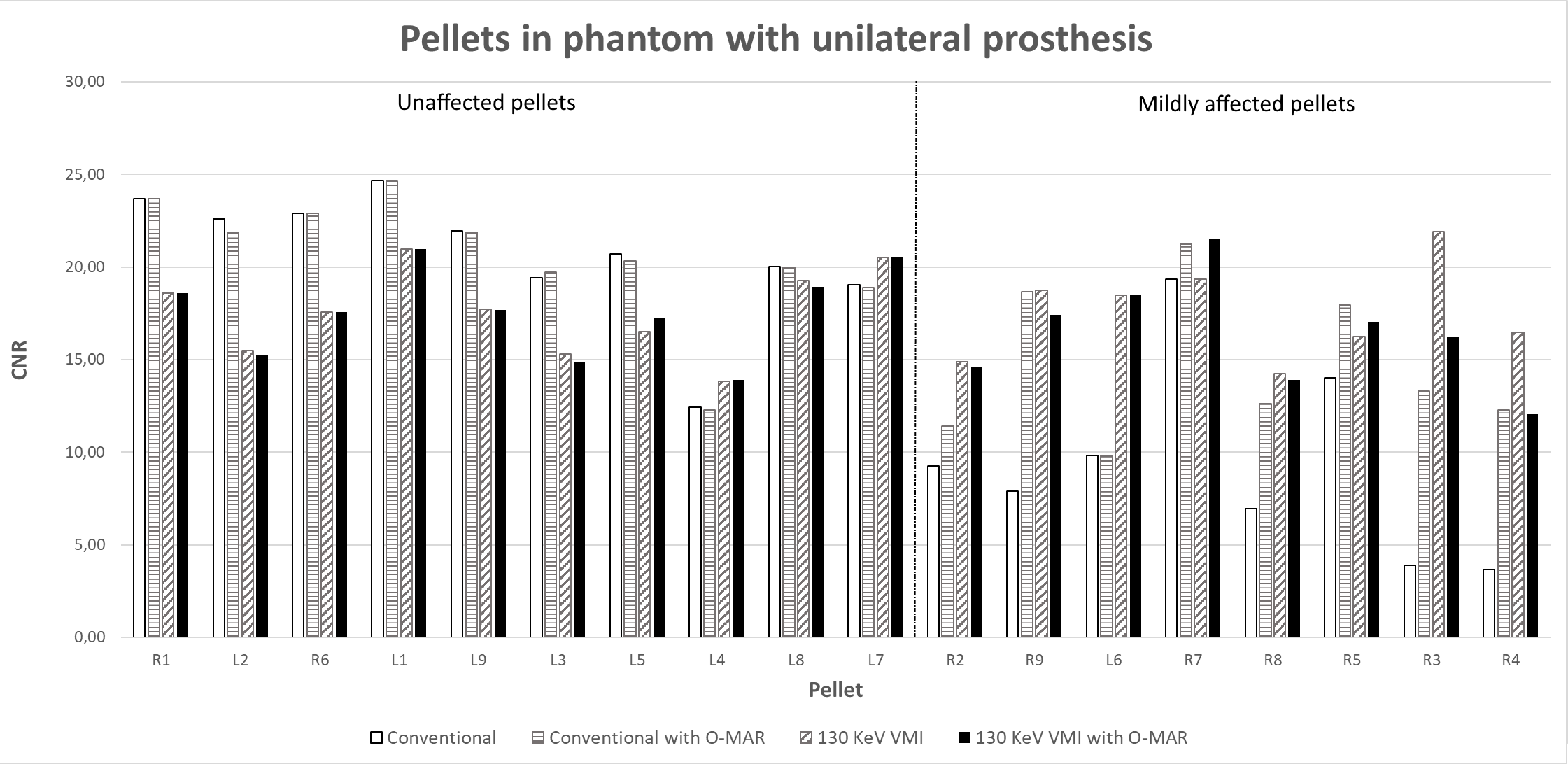


**Supplementary materials 2:** CNR of all pellets in the phantom with unilateral prosthesis. Unaffected pellets at the left, mildly affected pellets at the right.


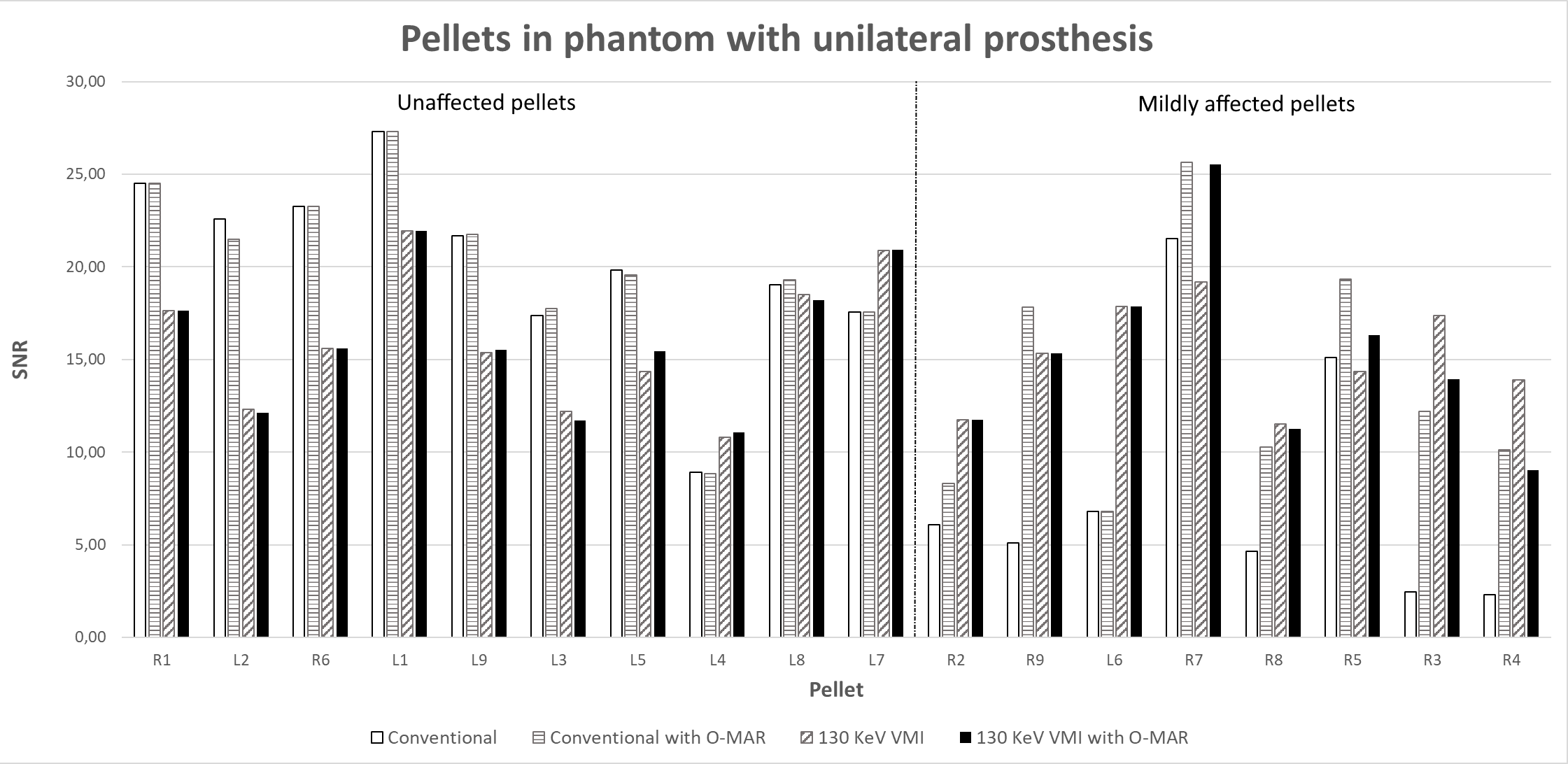
**Supplementary materials 3:** SNR of all pellets in the phantom with unilateral prosthesis. Unaffected pellets at the left, mildly affected pellets at the right.


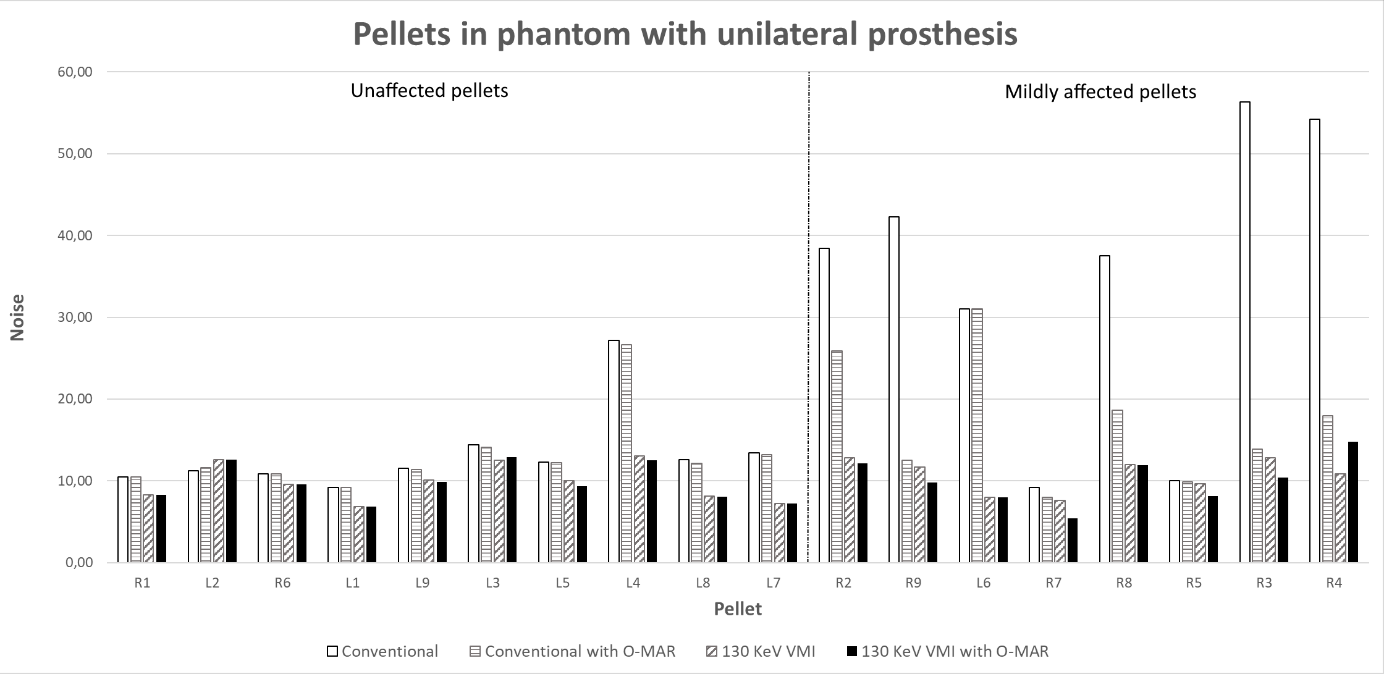


**Supplementary materials 4:** Noise of all pellets in the phantom with unilateral prosthesis. Unaffected pellets at the left, mildly affected pellets at the right.


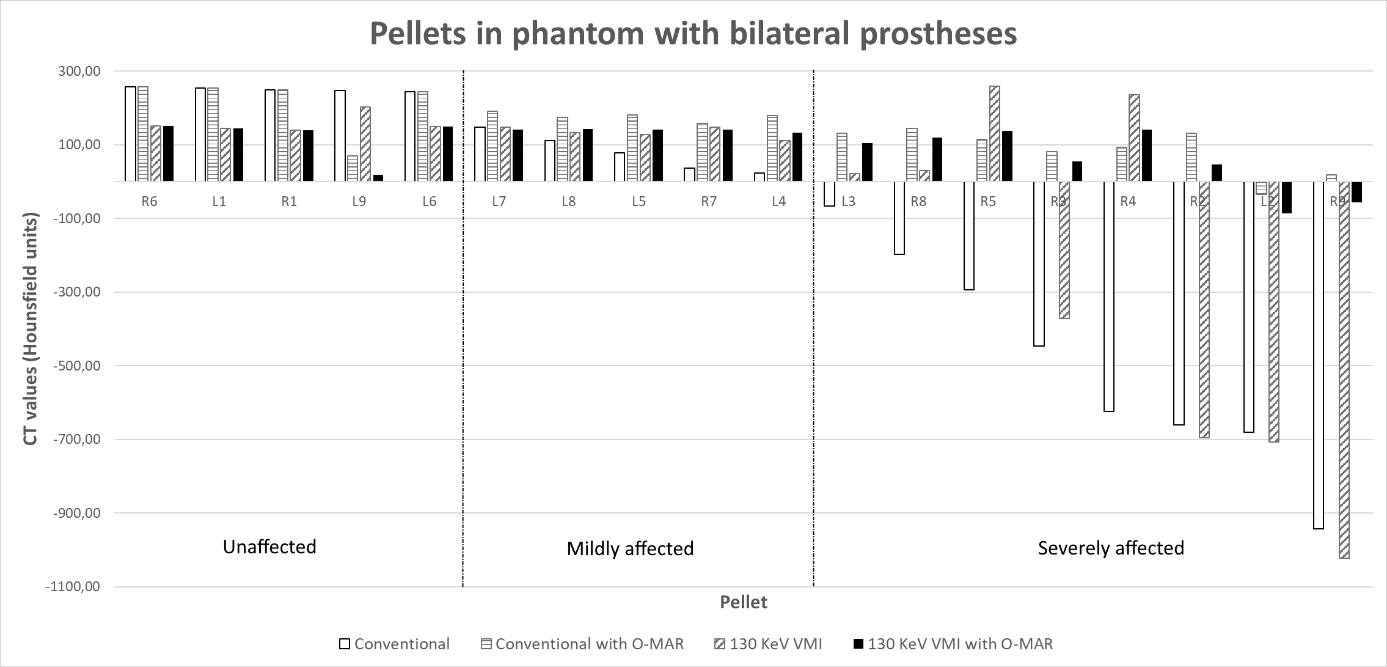
**Supplementary materials 5:** CT values in Hounsfield units of all pellets in the phantom with bilateral prosthesis. Unaffected pellets at the left, mildly affected pellets at the middle, and severely affected pellets at the right.


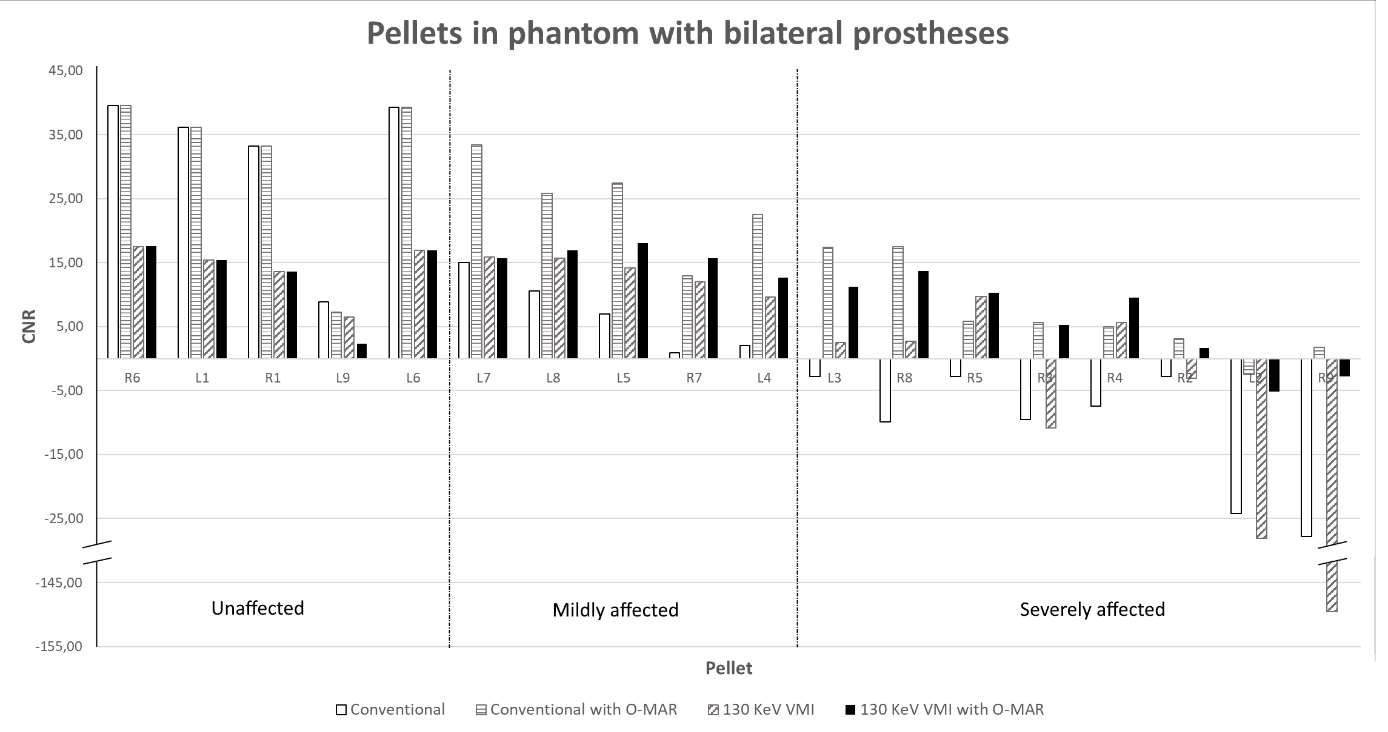


**Supplementary materials 6:** CNR of all pellets in the phantom with bilateral prosthesis. Unaffected pellets at the left, mildly affected pellets at the middle, and severely affected pellets at the right.


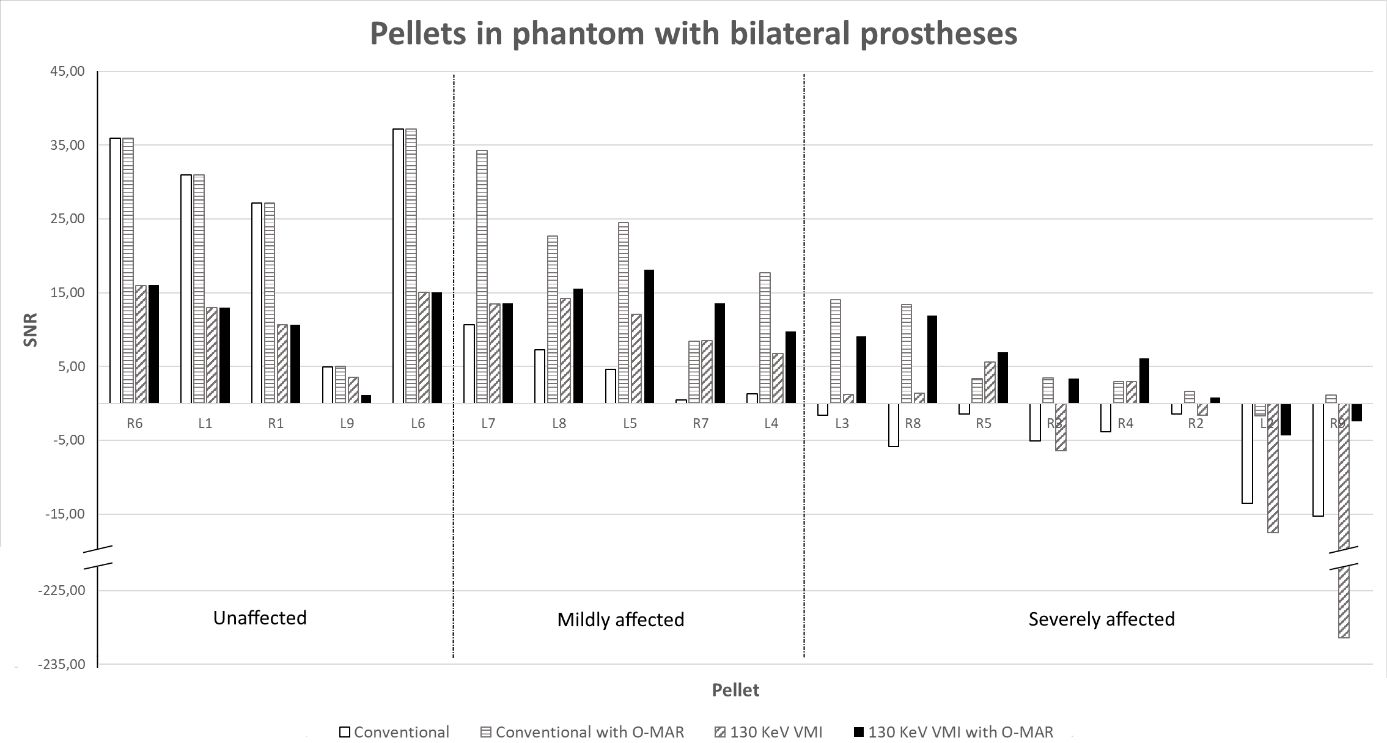


**Supplementary materials 7:** SNR of all pellets in the phantom with bilateral prosthesis. Unaffected pellets at the left, mildly affected pellets at the middle, and severely affected pellets at the right.


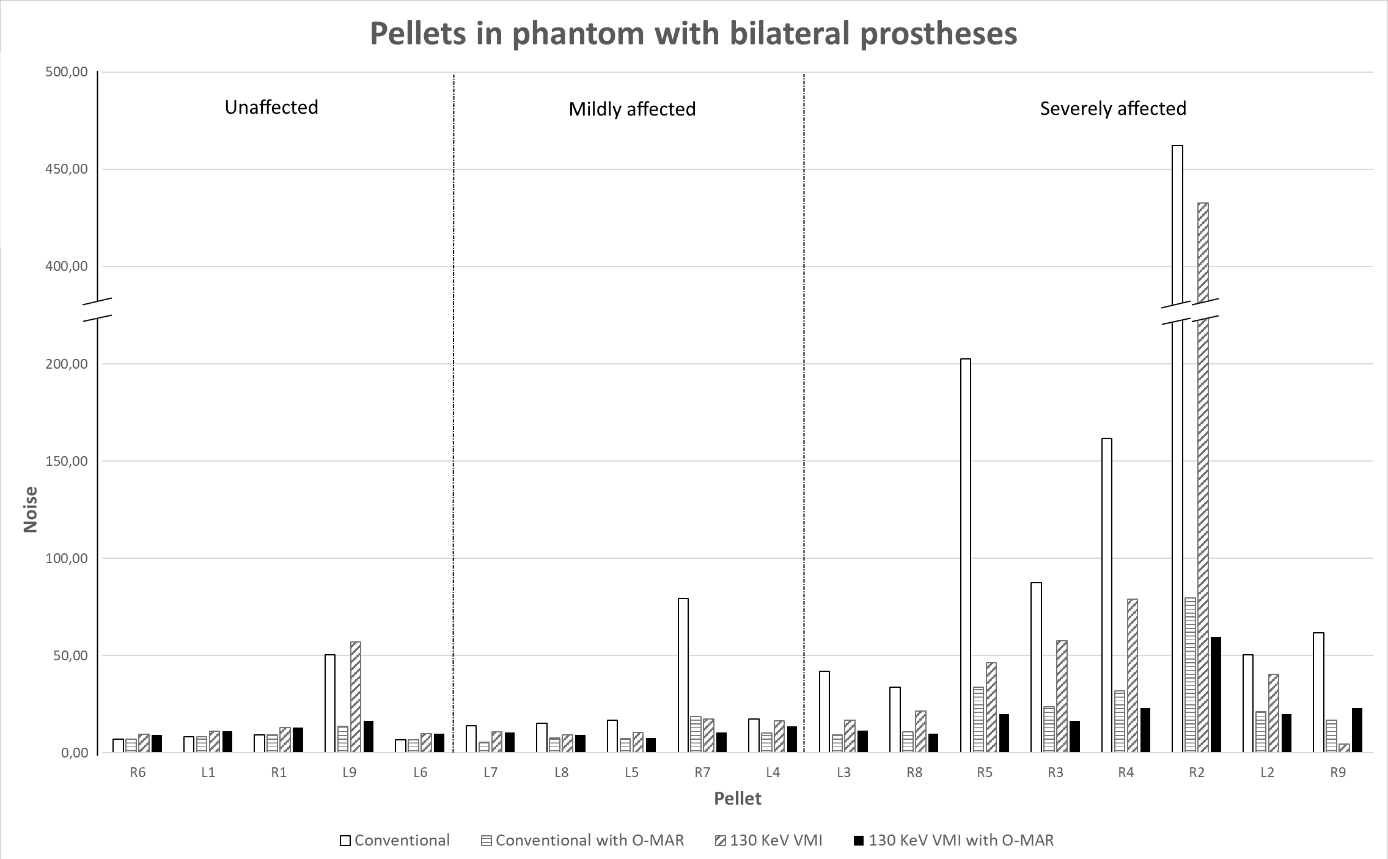


**Supplementary materials 8:** Noise of all pellets in the phantom with bilateral prosthesis. Unaffected pellets at the left, mildly affected pellets at the middle, and severely affected pellets at the right.
